# Second malignant neoplasms after a first cancer in childhood: temporal pattern of risk according to type of treatment

**DOI:** 10.1038/sj.bjc.6690300

**Published:** 1999-04

**Authors:** F de Vathaire, M Hawkins, S Campbell, O Oberlin, M-A Raquin, J-Y Schlienger, A Shamsaldin, I Diallo, J Bell, E Grimaud, C Hardiman, J-L Lagrange, N Daly-Schveitzer, X Panis, J-M Zucker, H Sancho-Garnier, F Eschwège, J Chavaudra, J Lemerle

**Affiliations:** Research Unit of Cancer Epidemiology (U351 INSERM), Department of Pediatrics, Radiotherapy, Institut Gustave Roussy, Villejuif, France; Childhood Cancer Research Group, Radcliffe Infirmary, Oxford, UK; Thames Cancer Registry, London, UK; Institut Jean Godinot, Reims, France; Centre Antoine Lacassagn, Nice, France; Centre Claudius Regaud, Toulouse, France; Institut Curie, Paris, France

## Abstract

The variation in the risk of solid second malignant neoplasms (SMN) with time since first cancer during childhood has been previously reported. However, no study has been performed that controls for the distribution of radiation dose and the aggressiveness of past chemotherapy, which could be responsible for the observed temporal variation of the risk. The purpose of this study was to investigate the influence of the treatment on the long-term pattern of the incidence of solid SMN after a first cancer in childhood. We studied a cohort of 4400 patients from eight centres in France and the UK. Patients had to be alive 3 years or more after a first cancer treated before the age of 17 years and before the end of 1985. For each patient in the cohort, the complete clinical, chemotherapy and radiotherapy history was recorded. For each patient who had received external radiotherapy, the dose of radiation received by 151 sites of the body were estimated. After a mean follow-up of 15 years, 113 children developed a solid SMN, compared to 12.3 expected from general population rates. A similar distribution pattern was observed among the 1045 patients treated with radiotherapy alone and the 2064 patients treated with radiotherapy plus chemotherapy; the relative risk, but not the excess absolute risk, of solid SMN decreased with time after first treatment; the excess absolute risk increased during a period of at least 30 years after the first cancer. This pattern remained after controlling for chemotherapy and for the average dose of radiation to the major sites of SMN. It also remained when excluding patients with a first cancer type or an associated syndrome known to predispose to SMN. When compared with radiotherapy alone, the addition of chemotherapy increases the risk of solid SMN after a first cancer in childhood, but does not significantly modify the variation of this risk during the time after the first cancer. © 1999 Cancer Research Campaign


*British Journal of Cancer* (1999) **79**(11/12), 1884–1893
© 1999 Cancer Research Campaign
Article no. bjoc.1998.0300

### Second malignant neoplasms after a first cancer in childhood: temporal pattern of risk according to type of treatment

**F de Vathaire[1], M Hawkins[4], S Campbell[5], O Oberlin[3], M-A Raquin[3], J-Y Schlienger[6], A Shamsaldin[1,3], I Diallo[1,3], J Bell[5], E Grimaud[3], C Hardiman[1,3], J-L Lagrange[7], N Daly-Schveitzer[8], X Panis[6], J-M Zucker[9], H Sancho-Garnier[1], F Eschwège[3], J Chavaudra[3] and J Lemerle[2]**

[1]Research Unit of Cancer Epidemiology (U351 INSERM), and Department of [2]Pediatrics and [3]Radiotherapy, Institut Gustave Roussy, Villejuif, France; [4]Childhood Cancer Research Group, Radcliffe Infirmary, Oxford, UK; [5]Thames Cancer Registry, London, UK; [6]Institut Jean Godinot, Reims, France; [7]Centre Antoine Lacassagne, Nice, France; [8]Centre Claudius Regaud, Toulouse, France; [9]Institut Curie, Paris, France

**Summary** The variation in the risk of solid second malignant neoplasms (SMN) with time since first cancer during childhood has been previously reported. However, no study has been performed that controls for the distribution of radiation dose and the aggressiveness of past chemotherapy, which could be responsible for the observed temporal variation of the risk. The purpose of this study was to investigate the influence of the treatment on the long-term pattern of the incidence of solid SMN after a first cancer in childhood. We studied a cohort of 4400 patients from eight centres in France and the UK. Patients had to be alive 3 years or more after a first cancer treated before the age of 17 years and before the end of 1985. For each patient in the cohort, the complete clinical, chemotherapy and radiotherapy history was recorded. For each patient who had received external radiotherapy, the dose of radiation received by 151 sites of the body were estimated. After a mean follow-up of 15 years, 113 children developed a solid SMN, compared to 12.3 expected from general population rates. A similar distribution pattern was observed among the 1045 patients treated with radiotherapy alone and the 2064 patients treated with radiotherapy plus chemotherapy; the relative risk, but not the excess absolute risk, of solid SMN decreased with time after first treatment; the excess absolute risk increased during a period of at least 30 years after the first cancer. This pattern remained after controlling for chemotherapy and for the average dose of radiation to the major sites of SMN. It also remained when excluding patients with a first cancer type or an associated syndrome known to predispose to SMN. When compared with radiotherapy alone, the addition of chemotherapy increases the risk of solid SMN after a first cancer in childhood, but does not significantly modify the variation of this risk during the time after the first cancer.

**Keywords:**

The temporal pattern of the occurrence of second primary malignant neoplasms after a cancer in childhood is of major clinical importance in determining the risk/benefit balance of different treatments. It plays a critical role in the estimation of the lifetime excess risk experienced by childhood cancer survivors.

Secondary leukaemias occurring after chemotherapy alone (Curtis et al, 1992; Hawkins et al, 1992) and their radiotherapy-induced counterparts, as well as those occurring after a combination of radiotherapy and chemotherapy (Tucher et al, 1987; Andrieu et al, 1990; Henry Amar and Dietrich, 1993; Olsen et al, 1993) appear to exhibit a wave-like temporal pattern, with most of the excess occurring within 10 years of the first cancer. Their temporal pattern of onset is not substantially different from that of leukaemias following irradiation for a non-malignant disease or for a non-medical reason (UNSCEAR, 1994).

A recent analysis of data on the Japanese atomic bomb survivors showed a significant decrease in the relative risk of solid cancer with follow-up after irradiation (UNSCEAR, 1994; Thompson et al, 1994). Although such a decline was not identified in previous analyses of this cohort (Pierce et al, 1991), it had been demonstrated by Little et al (1991, 1997) who pooled three cohorts including the atomic bomb survivors. Such a decrease has also been confirmed for breast cancer incidence among A-bomb survivors who were less than 20 years of age at the time of exposure (Tokunaga et al, 1994).

In contrast, the pattern of the variation of the risk of solid second malignancy after a first cancer in childhood is not well known. Indeed, the variation, or the absence of variation, of the risk observed in the major cohorts could be due to a real variation in the risk, or to an effect of the first cancer treatment of patients which varied with time (Tucker et al, 1984; Hawkins et al, 1987; Olsen et al, 1993). Patients with a longer follow-up were necessarily treated a longer time ago, and often according to protocols no longer used. Satisfactory control for such sources of variation in the risk of second malignant neoplasm (SMN) requires not only detailed information about amount and type of each chemotherapeutic drug, but also radiodosimetry to estimate the radiation dose delivered to each potential anatomical site of second cancer, for each child in the cohort.

We present here the results of a cohort study of 4400 3-year survivors of a childhood cancer monitored in order to evaluate the

*Received 6 March 1998*
*Revised 23 September 1998*
*Accepted 1 October 1998*

*Correspondence to:* F de Vathaire, Unité de Recherche en Epidémiologie des Cancers, INSERM U351, Institut Gustave Roussy, Rue Camille Desmoulins, 94805 Villejuif, Cedex, France

**Table 1** General characteristics of the cohort of 4400 3-year survivors of a first cancer in childhood

| First cancer | Patients | Mean year of first cancer treatment | Females (%) | Mean age at first cancer (years) | Mean follow-up (years) | Type of the first cancer treatment | | |
|---|---|---|---|---|---|---|---|---|
| | | | | | | Rt, no Ct (%) | Ct, no Rt (%) | Rt + Ct (%) |
| Ewing's sarcoma | 148 | 1976 | 36 | 9 | 12 | 9 | 5 | 86 |
| Bone sarcoma | 143 | 1977 | 42 | 12 | 12 | 23 | 37 | 29 |
| Soft tissue sarcoma | 588 | 1974 | 45 | 6 | 15 | 17 | 21 | 49 |
| Neuroblastoma | 566 | 1975 | 50 | 2 | 15 | 16 | 31 | 40 |
| Wilm's tumour | 816 | 1973 | 47 | 3 | 16 | 11 | 24 | 62 |
| Central nervous system | 722 | 1972 | 51 | 7 | 16 | 59 | 1 | 25 |
| Bilateral retinoblastoma | 82 | 1978 | 49 | 1 | 12 | 29 | 1 | 67 |
| Unilateral retinoblastoma | 59 | 1977 | 45 | 3 | 13 | 25 | 18 | 39 |
| Hodgkin's disease | 374 | 1975 | 35 | 10 | 13 | 23 | 8 | 68 |
| Non-Hodgkin's lymphoma | 456 | 1977 | 28 | 8 | 12 | 9 | 39 | 51 |
| Others | 446 | 1975 | 51 | 7 | 15 | 28 | 25 | 28 |
| Total | 4400 | 1974 | 45 | 6 | 15 | 24 | 20 | 47 |

Rt, radiotherapy; Ct, chemotherapy.

**Table 2** Treatment characteristics for 3109 children who received radiotherapy, and mean radiation dose to some selected organs for 2831 patients for whom dose estimation was possible

| First cancer | Radiotherapy (*n* = 3109) | | | | External radiotherapy with reconstruction of the dosimetry (*n* = 2831) | | | | | | | | | |
|---|---|---|---|---|---|---|---|---|---|---|---|---|---|---|
| | Total (*n*) | Brachytherapy (*n*) | External radiotherapy | | Type[a] | | | | Mean no. of fractions | Mean radiation dose in Gy | | | | |
| | | | Dosimetry not possible (*n*) | Dosimetry possible (*n*) | rx low energy (*n*) | Cobalt (*n*) | rx high energy (*n*) | e-(*n*) | | Cible volume (tumour) | Brain | Thyroid | Breasts | Digestive tract |
| Ewing's sarcoma | 141 | 1 | 26 | 114 | 4 | 89 | 30 | 17 | 26 | 74 | 1.8 | 2.8 | 5.6 | 7.1 |
| Bone sarcoma | 74 | 0 | 10 | 64 | 0 | 53 | 12 | 3 | 23 | 67 | 0.3 | 2.7 | 8.0 | 5.4 |
| Soft tissue sarcoma | 390 | 51 | 41 | 298 | 40 | 207 | 38 | 55 | 20 | 62 | 5.4 | 5.7 | 3.1 | 6.2 |
| Neuroblastoma | 317 | 0 | 15 | 302 | 66 | 168 | 29 | 54 | 16 | 34 | 2.3 | 3.5 | 5.5 | 8.4 |
| Wilm's tumour | 599 | 1 | 19 | 579 | 135 | 373 | 105 | 7 | 19 | 35 | 0.6 | 2.7 | 7.2 | 11.9 |
| Central nervous system | 604 | 9 | 19 | 576 | 86 | 383 | 145 | 89 | 29 | 74 | 25.3 | 6.2 | 2.0 | 3.9 |
| Bilateral retinoblastoma | 79 | 8 | 2 | 69 | 3 | 18 | 23 | 34 | 21 | 52 | 10.1 | 0.9 | 0.7 | 0.5 |
| Unilateral retinoblastoma | 36 | 2 | 1 | 33 | 5 | 12 | 1 | 16 | 24 | 49 | 8.6 | 2.2 | 1.6 | 1.4 |
| Hodgkin's disease | 342 | 1 | 19 | 322 | 24 | 147 | 179 | 31 | 24 | 61 | 3.3 | 23.4 | 11.1 | 13.4 |
| Non-Hodgkin's lymphoma | 274 | 2 | 8 | 264 | 25 | 204 | 46 | 19 | 17 | 42 | 14.6 | 7.0 | 3.1 | 5.1 |
| Others | 253 | 21 | 22 | 210 | 29 | 153 | 46 | 24 | 23 | 66 | 5.1 | 9.4 | 4.9 | 12.2 |
| Total | 3109 | 96 | 182 | 2831 | 417 | 1807 | 654 | 349 | 22 | 55 | 8.6 | 7.0 | 5.1 | 8.1 |

[a]Patients may have been treated with more than one type of machine.

long-term risk of SMN after a first cancer in childhood. A preliminary 'note' concerning some of these children, those treated by radiotherapy alone, has been published (de Vathaire et al, 1995). In the present report, we compare the variation of the risk with time since first cancer, according to the type of treatment, with emphasis on the comparison between radiotherapy alone and radiotherapy plus chemotherapy.

### PATIENTS AND METHODS

### Patients

A cohort of 4400 children treated in eight centres in France and the UK was established comprising patients who were alive 3 years after the first cancer, diagnosed before the age of 17 years and before 1986. All patients fulfilling these criteria in

© Cancer Research Campaign 1999

each participating centre were included. In British centres, patients with retinoblastoma were not included because they were the subject of a specific study (Hawkins et al, 1996). In both French and British centres, patients with leukaemia were not included (as either a first or a second cancer).

Clinical and histopathological characteristics of the first and second cancers, type of treatment, detailed information on chemotherapy, follow-up data and medical information about second cancers, were recorded from hospital clinical records by physicians or by a specialized data manager (British centres). Radiotherapy data were obtained from hospital technical radiotherapy records by physicians or hospital physicists. The mean year of first cancer treatment was 1974 (Table 1). The end-point of the cohort analysis was 1 January 1992 for patients treated in French centres and 1 January 1991 for those treated in British centres.

In 1995, a primary publication concerning only patients treated with radiotherapy alone (de Vathaire et al, 1995), dealt with 1055 patients and 26 solid SMN. Since this publication a. Eight patients who had been defined by mistake as in the ‘radiotherapy alone’ or in the ‘radiotherapy + chemotherapy’ group, have been reclassified in their true group of treatment.
b. We decided to keep only the 3-year survivors in the analysis, rather than 2-year survivors as in the previous publication, because the British patients were 3-year survivors
c. About a year of average follow-up was added.

### Radiation dosimetry

Of the 4400 patients, 3109 received radiotherapy (Rt). Dose estimation was not performed for the 96 patients who received brachytherapy. These 96 patients were excluded from the dose–response study. Among the 3013 other patients, dose estimation was not possible for 182 patients due to insufficient information, radiotherapy outside of a study treatment centre or irradiation of arms for which the positioning was unclear (Table 2). For each of the remaining 2831 patients who had received radiotherapy, absorbed radiation doses were estimated at 151 points of the body.

The individual dose calculation required for this study was performed with a software package, Dos_EG, which was developed for retrospective studies at the Institut Gustave Roussy (IGR) (Grimaud et al, 1994; Diallo et al, 1996; Shamsaldim et al, 1998). This software is based on auxological methods (Sempé, 1979) and is based on a previous model developed at IGR (François et al, 1988*a*, 1988*b*), in that:

i. the individual phantom is articulated, thus allowing for trunk inclination and back extension of the head as for mantle treatments
ii. the parameters used to adapt the generated phantom to the patient are increased to 12, allowing for better adaptation
iii. it localizes 151 anatomical sites using a Cartesian co-ordinate system instead of 64 as in the previous one.

For more than 50% of patients appropriate anatomical data (height, anterior–posterior thickness and left–right width) were available. For the others the standard anatomical dimensions of the French population corresponding to the same sex and age were used. For all patients the radiotherapy data mentioned above were available. For most of the radiotherapy machines used for this population, appropriate measurements were introduced to the software Dos_EG and used in dose calculations. For the others (ten of the 38 machines) the characteristics of similar machines at other centres were used.

Table 2 describes the characteristics of the treatment of the 3013 patients who received only external radiotherapy and the mean radiation dose delivered to some selected organs.

### Chemotherapy measurement

Chemotherapy (Ct) doses could not be found for 104 of the 2949 patients who had received chemotherapy (Table 3). Drugs were

**Table 3** Treatment characteristics for 2949 children who received chemotherapy, and mean number of moles m$^{-2}$ by type of drug for 2845 patients for whom chemotherapy reconstruction was possible

| First cancer | Chemotherapy | | All drugs (*n* = 2845) | | Electrophiles agents (*n* = 2050) | | Topoisomerase II inhibitors (*n* = 2132) | | Inhibitors of nucleotide synthesis (*n* = 605) | | Spindle inhibitors (*n* = 2438) | |
|---|---|---|---|---|---|---|---|---|---|---|---|---|
| | Total (*n*) | Reconstruction not possible (*n*) | Mean number of agents | Mean number of moles m$^{-2}$ | % | Mean number of moles m$^{-2}$ | % | Mean number of moles m$^{-2}$ | % | Mean number of moles m$^{-2}$ | % | Mean number of moles m$^{-2}$ |
| Ewing's sarcoma | 134 | 2 | 4.0 | 71 | 91 | 76 | 83 | 1.61 | 5 | 9.7 | 83 | 0.035 |
| Bone sarcoma | 94 | 5 | 3.1 | 113 | 56 | 29 | 59 | 0.55 | 57 | 169.9 | 30 | 0.026 |
| Soft tissue sarcoma | 414 | 8 | 2.7 | 30 | 64 | 46 | 69 | 0.49 | 4 | 9.7 | 64 | 0.032 |
| Neuroblastoma | 403 | 18 | 2.1 | 23 | 67 | 32 | 35 | 1.31 | 2 | 72.6 | 65 | 0.022 |
| Wilm's tumour | 701 | 17 | 1.8 | 4 | 9 | 42 | 81 | 0.18 | 1 | 1.1 | 64 | 0.031 |
| Central nervous system | 184 | 9 | 0.6 | 4 | 20 | 14 | 3 | 1.00 | 5 | 30.3 | 22 | 0.027 |
| Bilateral retinoblastoma | 56 | 11 | 1.7 | 17 | 60 | 28 | 36 | 0.21 | 2 | 0.2 | 54 | 0.012 |
| Unilateral retinoblastoma | 32 | 3 | 1.4 | 16 | 52 | 31 | 30 | 0.07 | 2 | 0.3 | 46 | 0.014 |
| Hodgkin's disease | 286 | 15 | 3.0 | 26 | 70 | 36 | 21 | 1.88 | 2 | 5.6 | 72 | 0.097 |
| Non-Hodgkin's lymphoma | 407 | 5 | 5.8 | 156 | 84 | 41 | 80 | 0.82 | 79 | 149.1 | 86 | 0.030 |
| Others | 238 | 11 | 1.9 | 12 | 40 | 24 | 44 | 0.50 | 17 | 12.0 | 37 | 0.033 |
| Total | 2949 | 104 | 2.4 | 35 | 48 | 38 | 49 | 0.64 | 14 | 115.9 | 57 | 0.036 |

 *© Cancer Research Campaign 1999*

**Table 4** Number of patients, observed and expected number of SMN, 25 years cumulative incidence[a], excess absolute risk (EAR) and relative risk (RR) of solid SMN, by type of first cancer

| First cancer | Solid SMN | | | | |
|---|---|---|---|---|---|
| | O | E | 25-year incidence (%) and 95% CI | EAR/$10^4$ PY (95% CI) | $RR^+$ (95% CI) |
| Ewing's sarcoma | 11 | 0.37 | 10 (3–16) | 75 (38–131) | 30 (15–51) |
| Bone sarcoma | 1 | 0.46 | 2 (0–5) | 5 (< 0–29) | 2 (0–10) |
| Soft tissue sarcoma | 22 | 1.63 | 7 (4–11) | 29 (18–44) | 13 (9–20) |
| Neuroblastoma | 10 | 1.07 | 3 (1–6) | 11 (4–22) | 9 (5–16) |
| Wilm's tumour | 11 | 2.06 | 3 (1–6) | 8 (3–15) | 5 (3–9) |
| Central nervous system | 15 | 2.80 | 3 (1–5) | 14 (7–23) | 5 (3–9) |
| Bilateral retinoblastoma | 7 | 0.09 | 13 (2–24) | 93 (39–181) | 80 (35–155) |
| Unilateral retinoblastoma | 0 | 0.08 | 0 | – | – |
| Hodgkin's disease | 13 | 1.08 | 7 (2–12) | 29 (14–49) | 12 (7–20) |
| Non-Hodgkin's lymphoma | 12 | 1.00 | 10 (3–17) | 24 (12–43) | 12 (6–20) |
| Others | 11 | 1.70 | 4 (1–6) | 18 (8–32) | 6 (3–11) |
| Total | 113 | 12.35 | 4.9 (3.1–5.9) | 19 (15–23) | 9.2 (7.6–11) |

[a]First 3 years of follow-up were not taken into account. O: observed number of SMN, E: Expected number of SMN.

grouped into five classes according to their known mechanism of action in the cell: electrophil agents, spindle inhibitors, inhibitors of nucleotide synthesis, topoisomerase II inhibitors and other drugs. In order to quantify the total amount of drug administered in each class, we chose to convert the dose of each cytotoxic agent to moles per square meter, rather than to milligrams per square meter. However, we also analysed the data assuming equivalent carcinogenic effect per mg $m^2$ to ascertain whether this affected our conclusions.

### Statistical analysis

In order to estimate the expected number of cancers, by sex, 5-year calendar period and 5 years of attained age, we used data from the Danish Cancer Registry (Parkin et al, 1992). This registry was chosen because French registries did not cover the study period, and because differences in cancer incidence in Europe are very small for those under 45 years of age (Parkin et al, 1993), the maximal age attained by only 1% of our patients during the follow-up period. Another reason is that Danish data have been used in many other European studies. The expected number of cancers was obtained for each sex, 5-year age group and 5-year calendar period, by multiplying the incidence rate observed in Denmark by the number of person-years (PY) at risk.

With the exception of total body irradiation, radiotherapy delivers extremely heterogeneous irradiation with different parts of the body receiving very different doses. The concept of an average whole body dose was therefore considered unsatisfactory. The temporal pattern of occurrence of brain, breast and thyroid cancer was hence analysed adjusting for, respectively, dose to brain (nine anatomical points), breast (two anatomical points) and thyroid (three anatomical points). This type of analysis was not possible for bone, soft tissue sarcomas and skin cancers, because the anatomical sites vary are widely distributed over the body.

The ratio between the observed number and the expected number of SMN was the relative risk (RR) and was modelled assuming that the number of SMN followed a Poisson distribution (Breslow and Day, 1987). Similarly, the excess absolute risk (EAR) was defined as the difference between the observed and the expected number of SMN, and expressed in relation to the number of PY at risk. Statistical tests were performed by comparing the deviance of nested models (Breslow and Day, 1987).

The analysis was done stratifying for the first cancer type, except when the aim of the analysis was to check a predisposition for a given type of second cancer to develop after a given first cancer, in which case the analysis was performed by introducing an indicator variable for first cancer type in the log-linear term. AMFIT Software was used for these analyses (Preston et al, 1993). Ninety-five percent confidence intervals (95% CI) for parameters were estimated using maximum likelihood (Moolgavkar and Venzon, 1987).

A debate exists concerning the best way to take into account the variation in the risk of cancer with time: time since first cancer or attained age (Kellerer and Barclay, 1992). We were not able to contribute to this debate. Indeed, in our cohort, all patients were younger than 15 years old at the time of irradiation, and thus the time since first cancer and the attained age were too closely related. Hence we reported variations in EAR in of RR according to these two time scales.

### RESULTS

Before the end of the study, 532 (12%) of the 4400 children were lost to follow-up and 578 (13%) had died. The mean follow-up was 15 years (range 3–48 years) and 1062 children (24%) were followed up 20 years or more. A total of 113 patients (2.6%) developed a solid SMN (all SMN but leukaemias), after excluding non-melanoma skin cancer. The cumulative incidence of solid

© Cancer Research Campaign 1999

**Table 5** Relative risk (RR) and excess absolute risk (EAR) per $10^{-4}$ person-years of solid SMN by type of first cancer and time since the first cancer

| First cancer type | 3–9 years after first cancer | | 10–19 years after first cancer | | 20–29 years after first cancer | | 30 years or more after first cancer | |
|---|---|---|---|---|---|---|---|---|
| | RR[a] (O) | EAR[a] | RR (O) | EAR | RR (O) | EAR | RR (O) | EAR |
| Ewing's sarcoma | 55 (6) | 75 | 20 (3) | 59 | 20 (2) | 142 | 0 | – |
| Bone sarcoma | 0 | | 8 (1) | 20 | 0 | – | 0 | – |
| Soft tissue sarcoma | 27 (10) | 27 | 15 (8) | 29 | 8 (4) | 42 | 0 | – |
| Neuroblastoma | 3 (1) | 2 | 9 (3) | 10 | 15 (5) | 57 | 10 (1) | 99 |
| Wilm's tumour | 5 (2) | 3 | 9 (6) | 12 | 4 (3) | 15 | 0 | – |
| Central nervous system | 16 (7) | 15 | 7 (6) | 15 | 2 (2) | 4 | 0 | – |
| Bilateral retinoblastoma | 75 (3) | 61 | 140 (3) | 15 | 0 | – | 100 (1) | 955 |
| Unilateral retinoblastoma | 0 | – | 0 | – | 0 | – | 0 | – |
| Hodgkin's disease | 18 (6) | 24 | 10 (5) | 31 | 9 (2) | 56 | 0 | – |
| Non-Hodgkin's lymphoma | 15 (5) | 17 | 14 (5) | 33 | 11 (2) | 63 | 0 | – |
| Others | 15 (5) | 17 | 10 (5) | 24 | 0 | – | 2 (1) | 36 |
| Total | 16 (45) | 16 | 11 (45) | 21 | 6 (20) | 27 | 2 (3) | 16 |

[a] First 3 years of follow-up were not taken in account. O: observed number of SMN.

**Table 6** Relative risk (RR) and excess absolute risk (EAR) per $10^{-4}$ person-years of solid SMN by type of first cancer and attained age

| First cancer type | 3–9 years of age | | 10–19 years of age | | 20–29 years or age | | 30 years or more of age | |
|---|---|---|---|---|---|---|---|---|
| | RR[a] (O[a]) | EAR | RR (O) | EAR | RR (O) | EAR | RR (O) | EAR |
| Ewing's sarcoma | 0 | | 63 (5) | 71 | 25 (4) | 79 | 17 (2) | 150 |
| Bone sarcoma | 0 | | 0 | – | 5 (1) | 16 | 0 | |
| Soft tissue sarcoma | 0 | 7 | 41 (15) | 43 | 7 (4) | 19 | 3 (2) | 23 |
| Neuroblastoma | 4 (1) | 3 | 7 (2) | 5 | 15 (5) | 44 | 9 (2) | 91 |
| Wilm's tumour | 0 | | 9 (5) | 8 | 7 (5) | 18 | 2 (1) | 9 |
| Central nervous system | 14 (1) | 18 | 7 (8) | 17 | 3 (3) | 7 | 1 (2) | 5 |
| Bilateral retinoblastoma | 50 (2) | 70 | 150 (3) | 119 | 0 | 0 | 100 (1) | 867 |
| Unilateral retinoblastoma | 0 | | 0 | | 0 | 0 | 0 | |
| Hodgkin's disease | 0 | | 17 (4) | 19 | 10 (5) | 30 | 12 (4) | 88 |
| Non-Hodgkin's lymphoma | 25 (1) | 21 | 17 (5) | 19 | 11 (4) | 31 | 7 (2) | 63 |
| Others | 0 | | 28 (7) | 29 | 6 (3) | 16 | 1 (1) | 6 |
| Total | 7 (6) | 7 | 20 (54) | 21 | 8 (34) | 22 | 4 (17) | 31 |

[a]First 3 years of follow-up were not taken in account. O: Observed number of SMN.

SMN was 4.9%, (95% CI; 3.7–5.8%) 25 years after treatment of first cancer and 7.7% (95% CI; 5.0–8.2%) 30 years after (Table 4).

During the follow-up period, 12.9 solid SMN were expected in our cohort, leading to a RR of 9.2 (95% CI; 7.6–11), similar among males (RR = 10) and females (RR = 8). Each year, on average, the excess absolute risk of cancer among these survivors was 188 cases per $10^5$ PY (95% CI; 152–230) (Table 4). Both the RR and EAR of SMN were significantly higher among patients treated for bilateral retinoblastoma ($P$ < 0.0001) and Ewing's sarcoma ($P$ < 0.001) as the first cancer than for other cancers (Table 4).

The RR of SMN decreased with the time since first cancer, from 16 (95% CI; 12–21) between 3 and 9 years after the first cancer to 11 (95% CI; 8–15) between 10 and 19 years, 6 (95% CI; 3–8) between 20 and 29 years, and 1.7 (95% CI; 0.5–4) after 30 years or more (Table 5). This gradual decrease was significant ($P$ < 0.001) after adjustment for sex, age at diagnosis, first cancer type, radiotherapy (yes/no) and the total number of moles of all electrophil agents. The EAR of solid SMN increased non-significantly ($P$ = 0.1) with time after the first cancer (Table 5): 157 cases per $10^5$ PY (95% CI; 112–211) 3–9 years after the first cancer, 214 (95% CI; 151–290) 10–19 years after, 269 (95% CI; 147–433) 20–29 years after, and 161 cases per $10^5$ PY (95% CI; 0–560) 30 years after or more. We were not able to show a significant interaction ($P$ = 0.2) between the type of first cancer and the

 *© Cancer Research Campaign 1999*

**Table 7** Observed (O) and expected (E) number of SMN by type of first cancer

| First cancer | Solid SMN | | | | | | |
|---|---|---|---|---|---|---|---|
| | Bone O (E) | Soft-tissue O (E) | Brain O (E) | Thyroid O (E) | Breast O (E) | Melanoma O (E) | Others O (E) |
| Ewing's sarcoma | 8 (0.014) | 3 (0.009) | 0 (0.010) | 0 (0.007) | 0 (0.032) | 0 (0.034) | 0 (0.265) |
| Bone sarcoma | 0 (0.014) | 0 (0.010) | 0 (0.015) | 1 (0.008) | 0 (0.045) | 0 (0.042) | 0 (0.327) |
| Soft tissue sarcoma | 11 (0.064) | 4 (0.041) | 2 (0.055) | 2 (0.032) | 2 (0.153) | 0 (0.145) | 2 (1.141) |
| Neuroblastoma | 1 (0.053) | 1 (0.032) | 1 (0.046) | 5 (0.020) | 2 (0.082) | 0 (0.077) | 0 (0.766) |
| Wilm's tumour | 1 (0.096) | 2 (0.058) | 1 (0.072) | 2 (0.042) | 1 (0.140) | 2 (0.170) | 2 (1.485) |
| Central nervous system | 2 (0.083) | 2 (0.058) | 5 (0.084) | 1 (0.052) | 1 (0.394) | 3 (0.265) | 1 (1.858) |
| Bilateral retinoblastoma | 5 (0.005) | 1 (0.003) | 0 (0.006) | 0 (0.001) | 0 (0.001) | 0 (0.004) | 1 (0.067) |
| Unilateral retinoblastoma | 0 (0.005) | 0 (0.003) | 0 (0.004) | 0 (0.001) | 0 (0.001) | 0 (0.004) | 0 (0.063) |
| Hodgkin's disease | 2 (0.040) | 2 (0.028) | 1 (0.033) | 2 (0.021) | 4 (0.061) | 0 (0.103) | 2 (0.797) |
| Non-Hodgkin's lymphoma | 3 (0.044) | 1 (0.028) | 2 (0.031) | 1 (0.018) | 1 (0.077) | 0 (0.080) | 5 (0.719) |
| Others | 1 (0.047) | 2 (0.033) | 0 (0.052) | 0 (0.032) | 2 (0.291) | 1 (0.158) | 6 (1.096) |
| Total | 34 (0.466) | 18 (0.303) | 12 (0.409) | 14 (0.234) | 13 (1.277) | 6 (1.081) | 19 (8.583) |

**Table 8** Number of patients, type of treatment, observed number of SMN, mean age at SMN, excess absolute risk and relative risk of SMN, by type of SMN

| Site of the SMN | Patients | First cancer treatment (*n*) | | | | Mean age at SMN in years (range) | EAR[a]/10[5] PY (95% CI) | RR[a] (95% CI) |
|---|---|---|---|---|---|---|---|---|
| | | No Rt no Ct (%) | Rt no Ct (%) | Ct no Rt (%) | Rt & Ct (%) | | | |
| Bone sarcoma | 34 | 0 | 4 (12%) | 2 (6%) | 28 (82%) | 15 (6–34) | 64 (44–87) | 73 (51–100) |
| Soft tissue sarcoma | 18 | 0 | 3 (17%) | 1 (6%) | 14 (78%) | 22 (11–34) | 33 (20–52) | 59 (36–91) |
| Thyroid | 14 | 0 | 7 (50%) | 0 | 7 (50%) | 22 (14–28) | 26 (14–42) | 60 (34–97) |
| Breast | 13 | 1 | 4 (31%) | 2 (15%) | 6 (46%) | 28 (12–38) | 41 (18–74) | 11 (6–18) |
| Brain | 12 | 0 | 8 (67%) | 1 (8%) | 3 (25%) | 17 (8–30) | 22 (12–38) | 29 (16–49) |
| Malignant melanoma | 6 | 0 | 2 (33%) | 1 (17%) | 3 (50%) | 23 (16–30) | 7 (0–19) | 6 (2–11) |
| Others | 16 | 2 (12%) | 3 (19%) | 1 (6%) | 10 (63%) | 23 (4–38) | 10 (0–26) | 2 (1–3) |

[a]First 3 years of follow-up were not taken in account. Rt, radiotherapy; Ct, chemotherapy.

pattern of the variations of the RR but, due to the small number of patients of each first cancer type, the power of the test was low. The decrease observed in the RR of solid SMN (Table 5) with time following Hodgkin's disease ($P = 0.2$) and neuroblastoma ($P = 0.1$), and in EAR of solid SMN with time following central nervous system tumours ($P = 0.2$) were not significant, but the number of cases is small. We were not able to find any evidence for an interaction between the type of first cancer treatment (Ct vs Rt vs Ct + Rt) on the pattern of the variations of the RR ($P = 0.8$) or of the EAR of SMN ($P = 0.3$).

When considering the attained age of the patients, rather than the time since first cancer (Table 6), results were similar: the RR of solid SMN decreased significantly with attained age ($P < 0.001$) and the EAR showed an increase that was just significant ($P = 0.04$). A significant interaction was found between the type of first cancer treatment and the pattern of the variation of the EAR ($P = 0.01$): the increase of the EAR with attained age was stronger among patients treated with Ct + Rt than among those treated by Rt alone.

The most frequent types of solid SMN were bone and soft tissue sarcomas, thyroid, breast and brain cancers (Tables 7 and 8). Bone and soft tissue sarcomas occurred mainly after the combination of Rt + Ct, whereas thyroid, brain and breast cancer occurred both after Rt, Ct or both.

Bone and soft tissue sarcoma RR and EAR significantly increased with radiotherapy (yes/no) ($P < 0.001$) and the dose of electrophil agents ($P < 0.001$). Of the 34 cases of bone sarcoma, only one occurred 20 years or more after the first cancer. Thyroid carcinoma RR and EAR significantly increased with radiation dose to the thyroid ($P < 0.001$), but not with chemotherapy ($P = 0.6$), and decreased with age at irradiation ($P = 0.001$). Breast cancer RR and EAR significantly increased with the radiation dose ($P = 0.05$) to the breasts and with the administration of chemotherapy ($P < 0.0001$). Of the 13 breast cancers, 12 occurred

© Cancer Research Campaign 1999

in women who had attained the age of 20 years or more. After Hodgkin's disease, the breast cancer RR was 76 (95% CI,24–177). Brain cancer RR increased significantly with average radiation dose to the brain ($P$ = 0.05) and non-significantly with antimetabolites ($P$ = 0.1), and significantly decreased with age at first cancer ($P$ = 0.05). Among the 11 brain cancers, five appeared 10 years or more after the first cancer.

### Neither radiotherapy nor chemotherapy

Three solid SMN occurred among the 406 patients treated by surgery alone, compared to 2.0 expected SMN (RR = 1.5, 95% CI, 0.6–4) (Tables 9 and 10). Two of these occurred among women: a breast cancer at the age of 12 years, 9 years after a rhabdomyosarcoma, and a malignant histocytosis at the age of 16 years, 9 years after a malignant melanoma. The other occurred in a man: a colorectal cancer at the age of 29 years, 26 years after a rhabdomyosarcoma.

### Radiotherapy without chemotherapy

Thirty-one patients developed a solid SMN among the 1045 treated with radiotherapy alone, compared to 5.6 expected (RR = 5.6, 95% CI = 3.8–7.8) (Table 9). The cumulative incidence of solid SMN was 3.9% (95% CI = 2.3–5.5), 25 years after radiotherapy for the first cancer (Figure 1). The sites of solid SMN were the brain (eight cases observed, 0.18 expected), thyroid (seven cases, 0.098 expected), breast (four cases, 0.79 expected), bone (four cases, 0.15 expected), soft tissue (three cases, 0.11 expected), skin melanoma (two cases, 0.52 expected), and lung cancer, bladder cancer and non-Hodgkin's lymphoma (one case each).

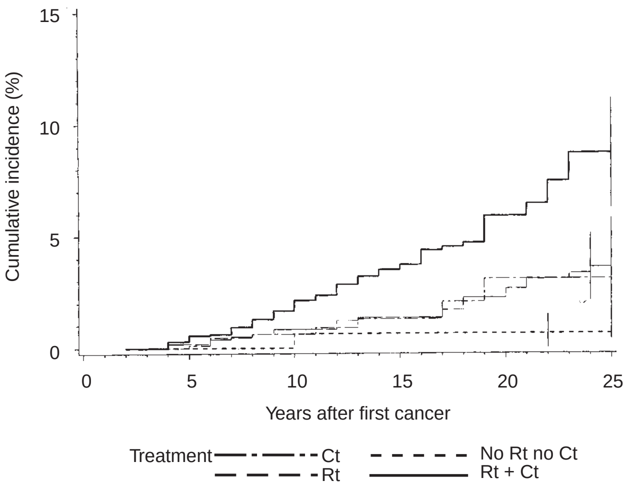

**Figure 1** Cumulative incidence of solid second malignant neoplasm and 95% CI. Rt, chemotherapy; Ct, chemotherapy

**Table 9** Solid SMN according to time since first cancer and to type of first cancer treatment

| Treatment and years after first cancer | Patients at risk at start of period | Solid SMN | | Annual incidence/10⁴ PY | | Relative risk (95% CI) |
|---|---|---|---|---|---|---|
| | | Observed | Expected | Total (95% CI) | Excess (95% CI) | |
| No Rt no Ct | 406 | 3 | 2.0 | 5 (1–12) | 3 (<0–10) | 2 (0.5–4.0) |
| 3–9 | 406 | 2 | 0.25 | 8 (1–25) | 7 (0.5–23) | 8 (1–25) |
| 10–19 | 308 | 0 | 0.54 | 0[a] | –0.5[a] | 0 (0–4) |
| 20–29 | 174 | 1 | 0.80 | 8 (0.5–36) | 2 (<0–32) | 1 (0.1–6) |
| ≥30 | 63 | 0 | 0.37 | 0 (<0–77) | –6 (<0–73) | 0 (0–5) |
| Rt no Ct | 1045 | 31 | 5.6 | 18 (12–25) | 15 (9–22) | 6 (4–8) |
| 3–9 | 1045 | 8 | 0.71 | 12 (6–22) | 11 (4–21) | 11 (5–21) |
| 10–19 | 831 | 11 | 1.59 | 17 (9–29) | 15 (6–26) | 7 (4–12) |
| 20–29 | 456 | 9 | 1.96 | 29 (14–52) | 23 (9–47) | 5 (2–8) |
| ≥30 | 177 | 3 | 1.31 | 35 (9–89) | 20 (<0–80) | 2 (0.6–6) |
| Ct no Rt | 885 | 8 | 1.1 | 11 (5–21) | 10 (3–19) | 8 (3–14) |
| 3–9 | 885 | 4 | 0.54 | 8 (2–18) | 7 (1–17) | 7 (2–17) |
| 10–19 | 380 | 3 | 0.33 | 16 (4–43) | 15 (2–41) | 9 (2–24) |
| 20–29 | 66 | 1 | 0.15 | 33 (2–146) | 28 (<0–140) | 7 (0.4–29) |
| ≥30 | 7 | 0 | 0.04 | 0 (<0–733) | –6[a] | 0 (0–27) |
| Rt and Ct | 2064 | 71 | 3.8 | 32 (25–40) | 30 (23–39) | 19 (15–24) |
| 3–9 | 2064 | 31 | 1.32 | 25 (17–35) | 24 (16–34) | 23 (16–33) |
| 10–19 | 1378 | 31 | 1.71 | 38 (26–53) | 36 (24–50) | 19 (12–25) |
| 20–29 | 366 | 9 | 0.70 | 62 (30–111) | 54 (23–104) | 13 (6–23) |
| ≥30 | 23 | 0 | 0.05 | 0 (<0–417) | –5 (<0–418) | 0[a] |

+ First 3 years of follow-up were not taken in account. Rt, radiotherapy; Ct, chemotherapy. [a]No convergence was obtained.

 © Cancer Research Campaign 1999

**Table 10** Solid SMN according to attained age and first cancer treatment

| Treatment and attained age | Patients at risk at start of period | Solid SMN | | Annual incidence/$10^4$ PY | | Relative risk (95% CI) |
|---|---|---|---|---|---|---|
| | | Observed | Expected | Total (95% CI) | Excess (95% CI) | |
| No Rt no Ct | 406 | 3 | 2.0 | 5 (1–12) | 3 (<0–10) | 2 (0.5–4.0) |
| 3–9 | 406 | 0 | 0.09 | 0 (0–17) | –0.5[a] | 0 (0–22) |
| 10–19 | 353 | 2 | 0.26 | 8 (1–25) | 7 (0.5–24) | 8 (1–24) |
| 20–29 | 252 | 1 | 0.56 | 5 (0.3–23) | 2 (<0–20) | 2 (0.1–7) |
| ≥30 | 124 | 0 | 1.01 | 0 (0–20) | –4[a] | 0 (0–2) |
| Rt no Ct | 1045 | 31 | 5.6 | 18 (12–25) | 15 (9–22) | 6(4–8) |
| 3–9 | 1045 | 3 | 0.15 | 14 (3–36) | 13 (3–35) | 19 (5–50) |
| 10–19 | 988 | 9 | 0.70 | 14 (6–24) | 12 (5–23) | 13 (6–23) |
| 20–29 | 730 | 10 | 1.79 | 18 (9–32) | 15 (6–29) | 6 (3–10) |
| ≥30 | 377 | 9 | 2.92 | 33 (16–60) | 25 (8–52) | 3 (1–6) |
| Ct no Rt | 885 | 8 | 1.1 | 11 (5–21) | 10 (3–19) | 8 (3–14) |
| 3–9 | 885 | 1 | 0.23 | 4 (0.2–17) | 3 (<0–16) | 4 (0.3–19) |
| 10–19 | 735 | 4 | 0.40 | 11 (4–26) | 10 (2–25) | 10 (2–23) |
| 20–29 | 228 | 2 | 0.29 | 22(4–66) | 18 (1–64) | 7 (1–24) |
| ≥30 | 30 | 1 | 0.14 | 66 (4–289) | 60 (<0–283) | 7 (0.4–31) |
| Rt and Ct | 2064 | 71 | 3.8 | 32 (25–40) | 30 (23–39) | 19 (15–24) |
| 3–9 | 2064 | 4 | 0.35 | 9 (3–20) | 8 (2–19) | 11 (4–26) |
| 10–19 | 1906 | 39 | 1.32 | 33 (24–45) | 32 (23–43) | 29 (21–40) |
| 20–29 | 1000 | 21 | 1.66 | 41 (26–61) | 38 (23–58) | 13 (8–19) |
| ≥30 | 166 | 7 | 0.43 | 128 (55–248) | 120 (46–239) | 16 (7–31) |

+ First 3 years of follow-up were not taken in account. Rt, radiotherapy; Ct; chemotherapy.[a] No convergence was obtained.

The mean annual incidence of solid SMN significantly increased from 120 (95% CI, 80–294) cases per $10^5$ PY 3–9 years after the first cancer to 345 cases per $10^5$ PY (95% CI, 86–894) 30 years or more later ($P < 0.0001$). The EAR reached a plateau 20 years after the first cancer, and the RR decreased from 11 (95% CI, 5–21) 3–9 years after the first cancer to 2.3 (95% CI, 0.6–5.9) 30 years or more after (Table 9) ($P < 0.0001$). A similar pattern was observed according to attained age (Table 10). These results have been detailed elsewhere (de Vathaire et al, 1995).

### Chemotherapy without radiotherapy

A total of eight patients developed a SMN after chemotherapy alone compared to 1.1 SMN expected (RR = 7.6, 95% CI = 3.5–14): two bone sarcomas (0.06 expected, RR = 32, 95% CI = 5.4–99), two breast cancers (0.05 expected, RR = 44, 95% CI = 7.3–135), brain cancer, soft tissue sarcoma, Hodgkin's disease and malignant histiocytosis (one case of each). The cumulative incidence of SMN 25 years after the first cancer was 2.7% (95% CI = 0.3–5.4). A temporal analysis was not possible because of the very short mean follow-up of 11 years (Tables 9 and 10).

### Both radiotherapy and chemotherapy

A total of 71 patients developed a solid SMN, compared to 3.8 expected (RR = 19, 95% CI = 15–24). The sites were the bone (28 cases observed, 0.20 expected, RR = 139, 95% CI = 94–197), soft tissue (14 cases, 0.12 expected, RR = 115, 95% CI = 65–185), thyroid (seven cases, 0.08 expected, RR = 86, 95% CI = 37–166), breast (six cases, 0.15 expected, RR = 40, 95% CI = 16–81), brain (three cases, 0.12 expected, RR = 26, 95% CI = 5.5–67), liver (three cases, 0.13 expected, RR = 9.3, 95% CI = 2.2–24) melanoma (three cases, 0.12 expected, RR = 9.9, 95% CI = 2.5–26), kidney (two cases, 0.15 expected, RR = 13, 95% CI = 0–33), and stomach, pancreas, multiple myeloma and parathyroid gland (one case each). The primary sites of a sarcoma and of a carcinoma were not localized. The 25-year cumulative incidence of solid SMN was 7.7% (95% CI = 5.4–9.9).

The RR of solid SMN significantly decreased with time since first cancer ($P < 0.05$) (Table 9) and with attained age ($P < 0.05$) (Table 10). This decrease remained significant ($P < 0.05$), when adjusted for age at first cancer, sex, country of treatment, first cancer type and the total number of moles of electrophil agents, and ($P < 0.05$) when patients known to be predisposed to SMN (five Recklinghausen, 14 Li–Fraumeni) were excluded. The same result remained significant ($P < 0.05$) when the analysis was restricted to the 1885 patients treated with external radiotherapy, for which the dose had been estimated, and when adjusted for the average dose of radiation received by the thyroid, brain, breast and digestive tract.

In contrast, the EAR of solid SMN significantly increased with time after irradiation ($P < 0.05$) and attained age ($P < 0.005$) from

240 (95% CI; 159–337) cases per $10^5$ PY between 3 and 9 years after the first treatment to 548 cases per $10^5$ PY (95% CI, 226–1039) 20 years or more after, this increase remaining significant after adjustment for treatment and first cancer type ($P < 0.01$ in all cases).

### DISCUSSION

This study of the incidence of solid second malignant neoplasms in a cohort of 4400 patients treated for a first cancer in childhood, and followed up for 15 years on average, revealed a significant increase in the annual excess incidence of a solid SMN ($P = 0.01$), and a decrease in the relative risk, with time since treatment of the first cancer, both for patients treated with radiotherapy alone ($P < 0.0001$) and for those treated with radiotherapy plus chemotherapy ($P < 0.05$), during a period of at least 30 years after the first cancer.

The reference cancer incidence registry we used did not cover the study area. This choice could have biased the estimation of the decrease in the RR if the Danish general population below 40 years of age had recently experienced a strong increase in cancer incidence, and if such an increase had not occurred in France and the UK. However, statistics published on cancer incidence do not show any sign of such a phenomenon (Parkin et al, 1993).

Among our cohort, 532 (12.1%) patients were lost to follow-up. These patients were not different from the others by sex (45% women) or diagnostic date (1975 on average). They were more frequently bilateral retinoblastoma (4% vs 2%) ($P = 0.09$). They received a lower number of moles of drug (22 vs 36 moles $m^{-2}$) ($P < 0.01$), a similar average radiation dose to thyroid (4.6 vs 4.4 Gy), to breast (3.2 vs 3.4 Gy), to stomach (5.4 vs 5.6 Gy) and to brain (7.6 vs 8.0 Gy).

The finding that the solid SMN RR decreased with time since first cancer and attained age, both after radiotherapy alone and after radiotherapy plus chemotherapy, is consistent with results concerning the overall risk of solid cancer after irradiation during childhood for other reasons (UNSCEAR, 1994; Little et al, 1997). This study also leads us to conclude that chemotherapy significantly increases the risk of solid cancer in patients treated with radiotherapy, but does not significantly affect the temporal pattern of the risk after the first cancer. Although other studies of SMN occurrence after first cancer were not able to control for the variation in the radiation dose distribution and in aggressiveness of the chemotherapy according to the period of treatment, our results may be compared with those of the other major studies. Our result concerning a significant decrease in solid SMN RR with time since treatment is in agreement with the finding of a study based on data from registries: a cohort of 30 880 patients treated in childhood or adolescence (Olsen et al, 1993). Two other cohort studies, comprising 10 106 3-year survivors followed 6 years in average (Tucker et al, 1984) and 9170 2-year survivors followed 8 years in average (Hawkins et al, 1987), found no evidence of a variation in the RR of SMN with time since the first cancer.

Excess absolute risk, which is of major clinical interest, was found to significantly increase with time since first cancer, at least during the initial 30 years after first cancer, and also with attained age. In our cohort, this increase was mainly due to breast and brain cancer. This result is in agreement with those of all the other cohort studies of SMN after first cancer in childhood (Tucker et al, 1984; Hawkins et al, 1987; Olsen et al, 1993). It was not due to a variation in the distribution of the radiation dose or in the aggressiveness of the chemotherapy according to the year of treatment. Indeed, it remained after controlling for these factors.

We found a relative risk of breast cancer after Hodgkin's disease of 76 (95% CI; 24–177), which is similar to the 75 (95% CI; 45–118) reported by Bhatia et al (1996) among 483 women treated for Hodgkin's disease during childhood. Although compatible, the cumulative risk of breast cancer at 40 years of age in our cohort was significantly lower than in this cohort (19% vs 35%). We found that the risk of thyroid cancer significantly increased with the dose of radiation, but not with chemotherapy, which is in agreement with the findings of Tucker et al (1991). The very high proportion (8/31) of second brain cancers among second cancers occurring in patients treated with radiotherapy alone was due to the high proportion of cancers of the central nervous system as the first cancer among patients treated with radiotherapy alone in our cohort (423/1046), particularly due to recruitment in British centres. These patients had received much higher doses to the brain and hence developed more second brain cancers of a different histological type. This higher risk totally disappeared after adjustment for the dose to the brain.

It has to be noted that patients in our cohort were young at the end of the follow-up: 21 years on average. Hence our study deals effectively only with solid SMN occurring during childhood or in young adults, and is not informative about solid SMN which will occur at later ages.

In conclusion, our study confirms that there is a high risk of second cancer occurring after radiotherapy and/or chemotherapy for a first cancer in childhood. On comparison with the general population, the relative risk decreased with time since the first cancer, but not the excess absolute risk, which increased during the period of at least 30 years after the first cancer. As compared to radiotherapy alone, the addition of chemotherapy increases the risk of SMN after a first cancer in childhood, but does not modify the variation of this risk during the time after the first cancer.

### ACKNOWLEDGEMENTS

This work was supported by special grants from the 'Radioprotection' and the 'Europe Against Cancer' Programs of the European Communities, Institut Gustave Roussy, Institut National de la Santé et de la Recherche Médicale (INSERM), Caisse Nationale Assurance Maladie des Travailleurs Sociaux (CNAMTS), Electricité de France (EDF), SNECMA, FRAMATOME, Association pour la Recherche sur le Cancer (ARC), and the comities 'Champagne Ardennes', 'Essonne' and 'Val d'Oise' of the Ligue Nationale Contre le Cancer (LNCC). This work was initiated by Maurice Tubiana, Odile Schweisguth, Robert Flamant, and Andrée Dutreix. We thank Armelle Leszczynski-Kramar, Gisèle Da Silva, Véronique Mosserie and Marie France Tournade for their help, and Lorna Saint Ange for editing the manuscript.

### APPENDIX

In addition to authors, the following medical doctors or physicists participated in the elaboration of the study or the data collection – Gustave Roussy Institute: Marta Guerra, Claire de Cervens, Aciera Suarez, Valérie Meresse, Pascal Pons, Nathalie Jan, Nathalie Rumeau, Ibrahima Diallo, Gilles Nicolazic, Annic Lamon; Insitut

 © Cancer Research Campaign 1999

Jean Godinot: Serge Théobald; Institut Curie: Geneviève Gaboriaud; Centre Claudius Regaud: Martine Roumagnac; Centre Antoine Lacassagne: Josiane Mercier-Waltzer.

### REFERENCES

Andrieu JM, Ifarh N, Payen C, Fermanian J, Coscas Y and Flandrin G (1990) Increased risk of secondary acute nonlymphocytic leukemia after extended-field radiation therapy combined with MOPP chemotherapy for Hodgkin's disease. *J Clin Oncol* **8**: 1148–1154

Bhatia S, Robison LL, Oberlin O, Greenberg M, Bunin G, Fossati-Bellani F and Meadows AT (1996) Breast cancer and other second neoplasms after childhood Hodgkin's disease. *N Engl J Med* **334**: 745–751

Blayney DW, Longo DL, Young RC and 5 others (1987) Decreased risk of leukemia with prolonged follow-up after chemotherapy and radiotherapy for Hodgkin's disease. *N Engl J Med* **316**: 710

Breslow NE and Day NE (1987) *Statistical Methods in Cancer Research. The Design and Analysis of Cohort Studies*. International Agency for Research on Cancer: Lyon

Curtis R, Boice JD, Stovall M and 7 others. (1992) Risk of leukemia after chemotherapy and radiation treatment for breast cancer. *N Engl J Med* **326**: 1745–1751

de Vathaire F, Shamsaldin A, Grimaud E and 26 others (1995) Solid malignant neoplasms after childhood irradiation: decrease of the relative risk with time after irradiation. *CR Acad Sci Paris* **318**: 483–490

Diallo I, Lamon A, Shamsaldin A, Grimaud E, de Vathaire F and Chavaudra J (1996) Estimation of the radiation dose delivered at any point in the body for individual patient in external beam radiotherapy. *Radiother Oncol* **38**: 269–278

François P, Beurtheret C, Dutreix A and de Vathaire F (1988*a*) A mathematical child phantom for the calculation of dose to the organs at risk. *Med Phys* **15**: 328–333

François P, Beurtheret C and Dutreix A (1988*b*) Calculation of the dose delivered to organs outside the radiation beams. *Med Phys* **15**: 879–883

Grimaud E, Shamsaldin A, Lamon A, Hardiman C, de Vathaire F and Chavaudra J (1994) Programme original de calcul de dose appliqué à l'étude de seconds cancers. *Bull Cancer Radiother* **81**: 482

Hawkins MM, Draper GJ and Kingston JE (1987) Incidence of second primary tumours among childhood cancer survivors. *Br J Cancer* **56**: 339–347

Hawkins MM, Kinnier Wilson LM, Stovall MA, Marsden HB, Potok MHN, Kingston JE and Chessells JM (1992) Epipodophyllotoxins, alkylating agents and radiation and risk of secondary leukemia after childhood cancer. *Br Med J* **304**: 951–958

Hawkins MM, Kinnier Wilson LM, Burton HS and 4 others (1996) Radiotherapy, alylating agents, and risk of bone cancer after a childhood cancer. *J Natl Cancer Inst* **88**: 270–278

Henry Amar M and Dietrich P-Y (1993) Acute leukemia after the treatment of Hodgkin's disease. *Hematol Oncol Clin North Am* **7**: 368–387

Kaldor JM, Day NE, Clark A and 25 others. (1990) Leukemia following Hodgkin's disease. *N Engl J Med* **322**: 7

Kellerer AM and Barclay D (1992) Age dependencies in the modeling of radiation carcinogenesis. *Radiat Prot Dosim* **41**: 273–281

Little MP, Hawkins MM, Shore RE and Hildreth NG (1991) Time variations in the risk following irradiation in childhood. *Radiat Res* **126**: 304–316

Little MP, de Vathaire F, Charles MW, Hawkins MM and Muirhead CR (1997) Variations with time and age in the relative risks of solid cancer incidence after radiation exposure. *J Rad Prot* **7**: 159–177

Moolgavkar SH and Venzon DJ (1987) A method for computing profile likelihood based confidence bounds. *Ann Statist* **15**: 346–359

Olsen JH, Garwicz S, Hertz H and 8 others (1993) Second malignant neoplasm after cancer in childhood or adolescence. *Br Med J* **307**: 1030–1036

Parkin DM, Muir CS, Whelan SL, Gao YT, Ferlay J and Powel J (1992) *Cancer Incidence in Five Continents, Vol VI*. International Agency for Research on Cancer: Lyon

Parkin DM, Muir CS, Whelan SL, Gao YT, Ferlay J and Powel J (1993) *Trends in Cancers Incidence and Mortality*. International Agency for Research on Cancer: Lyon

Pierce DA, Vaeth M and Preston D (1991) Analysis of time and age patterns in cancer risk for A-bomb surivors. *Radiat Res* **127**: 171–186

Preston DL, Lubin JH and Pierce DA (1993) *EPICURE User's Guide*. Hirosoft International Corporation: Seattle, WA

Sempé M (1979) *Auxologie, méthodes et séquences*. Theraplix: Paris

Shamsaldim A, Grimaud E, Hardiman C, Diallo I, de Vathaire F and Chavaudra J (1998) Dose distribution throughout the body for radiotherapy for Hodgkin's disease in childhood. *Radiother Oncol* (in press)

Thompson DE, Mabuchi K, Ron E and 8 others (1994) Cancer incidence in atomic bomb survivors. Part II: Solid tumors, 1958–1987. *Radiat Res* **137**: 17–67

Tokunaga M, Land CE, Tokuoka S, Nishimori I, Soda M and Akiba S (1994) Incidence of breast cancer among atomic bomb suvivors, 1950–1985. *Radiat Res* **138**: 209–223

Tucker MA, Meadow AT, Boice JD, Hoover RN and Fraumeni JF (1984) Cancer risk following treatment of childhood cancer. In *Radiation Carcinogenesis: Epidemiology and Biological Significance*, Boice JD and Fraumeni JF (eds), p. 211. Raven Press; New York

Tucker MA, Meadow AT, Boice JD and 11 others (1987) Leukemia after therapy with alkylating agent for childhood cancer. *J Natl Cancer Inst* **78**: 459–593

Tucker MA, Morris-Jones PH, Boice JD and 10 others (1991) Therapeutic radiation at a young age is linked to secondary thyroid cancer. *Cancer Res* **51**: 2885–2888

UNSCEAR (1994) *Sources, Effects and Risks of Ionizing Radiation*. United Nations: New York

© Cancer Research Campaign 1999 
